# Prediction of Prognosis in Patients with Hepatocellular Carcinoma Based on Molecular Subtypes of Immune Genes

**DOI:** 10.1155/2022/2746156

**Published:** 2022-06-28

**Authors:** Suming Du, Jinhui Xu, Jiajia Shen, Xiaojin Zhang, Huanzhang Hu, Xinghua Huang

**Affiliations:** Department of Hepatobiliary Surgery, The 900th Hospital of the Joint Logistic Support Force of People's Liberation Army, Fuzhou 350025, China

## Abstract

For those patients with hepatocellular carcinoma (HCC), it is really a heavy burden. Herein, the immune genes of HCC were analyzed in groups to determine prognostic biomarkers related to immune genes in HCC. The mRNA data, clinical data in TCGA-LIHC dataset, and immune gene in the ImmPort database were collected for the combining usage with *K*-means concordance clustering to cluster HCC patients according to the immune gene matrix. Based on ssGSEA analysis result, HCC patients were sorted into high- and low-immune subtypes, and survival curve presented that patients in high-immune subtypes had a better prognosis. Subsequently, differential expression analysis was performed to obtain immune-related differentially expressed genes (IRGs). Cox and lasso analyses were performed for obtaining five optimal immune genes related to prognosis, and a risk assessment model was then established. Patient samples in the training and validation sets were, respectively, divided into high- and low-risk groups. *K*-*M* survival curves presented a better prognosis of patients in the low-risk group than in the high-risk group. The ROC curve indicated that this model was finely used for the prediction of prognosis. In addition, immune infiltration assessment revealed that NR0B1 and FGF9 had potential to impact the tumor immune microenvironment. Finally, using qRT-PCR and transwell assays, it was demonstrated that the macrophage chemotaxis was enhanced when NR0B1 and FGF9 were highly expressed in HCC cells. In general, a 5-gene prognostic risk assessment model was constructed based on immune genes and bioinformatics analysis methods, which provides some reference for the prognosis of HCC as well as immunotherapy.

## 1. Introduction

Liver cancer is a primary cancer in the liver, and cancer arising from liver cells is called primary liver cancer. The World Health Organization (WHO) in 2020 ranks primary liver cancer as the sixth most frequent cancer throughout the world [1]. Hepatocellular carcinoma (HCC) makes up for 75%–85% of primary liver cancers [1]. Current treatment methods of HCC mainly include radiofrequency ablation, clinical surgery, liver transplantation, and neoadjuvant chemoradiotherapy, but the effect is not satisfactory, and only a few patients benefit from it. However, due to the insidious progression, poor therapeutic effect, and high recurrence rate, the overall prognosis of patients is poor [2]. Hence, there is an urgent need to find a biomarker closely related to HCC development and progression in order to better predict recurrence, metastasis, and prognosis.

In recent years, the clinical development and application of molecular targeted drugs and immunotherapeutic drugs have become a hot spot for the treatment of HCC [3]. Over the decades, immune checkpoint inhibitors (PD-1, CTLA-4, etc.) have shown good therapeutic effect as adjuvant therapies for tumors, and the approved immune checkpoint inhibitors have become new pillars of cancer care [4–6]. Camrelizumab is an anti-PD-1 monoclonal antibody, and apatinib represses vascular endothelial growth factor receptor (VGEFR) [7]. An open label phase II study demonstrated that camrelizumab combined with apatinib (treatment with camrelizumab by intravenous injection every 2 weeks and a certain amount of apatinib taken orally every day) had promising antitumor effect in HCC in first- and second-line settings [8]. A phase III clinical study is currently ongoing. El-Khoueiry et al. [9] demonstrated that nivolumab, a PD-1 immune checkpoint inhibitor, had a favorable safety profile in patients with HCC. The impact of cancer immunotherapy is closely related to the tumor immune microenvironment. Rapoport et al. [10] performed immunotherapy in 20 patients with advanced multiple myeloma, of whom 16 patients (80%) developed an immune response with TCR-T cells targeting NY-ESO-1. Shi et al. [11] revealed that the number of PD-1^+^ and CD8^+^ T cells within the tumor or in the circulation was positively associated with HCC progression and recurrence. Leukocyte infiltration around tumor vessels was also confirmed to be an independent risk factor for HCC patients' prognosis [12]. Consequently, constructing immune-related biomarkers based on expression of genes in tumor immune microenvironment is feasible.

On the basis of the TCGA database, mRNA data and immune genes of HCC were analyzed by bioinformatics methods, and a prognostic risk assessment model of HCC associated with immune genes was constructed and validated. Finally, the effects of NR0B1 and FGF9 on macrophage chemotaxis were examined in order to provide prognostic genes and immune targeted therapy in patients with HCC.

## 2. Material and Method

### 2.1. Data Downloadin and Preprocessing

HCC mRNA expression dataset TCGA-LIHC (normal: 50, tumor: 374) and its clinical data were acquired from the TCGA database (https://portal.gdc.cancer.gov/). And immune-related gene sets were obtained from The Immunology Database and Analysis Portal (ImmPort; https://www.immport.org/resources). Then, immune-related genes in HCC were extracted for subsequent studies (Table S1).

### 2.2. Immune Grouping and Survival Analysis

HCC samples were subjected to K-means concordance clustering analysis using the R package “ConsensusClusterPlus” [13]. The double sampling scheme was used, 80% of the samples were sampled each time, and 1000 times were set repeatedly. According to the CDF diagram, optimal number of clusters was determined, and stability of clustering results could be achieved when the number of clusters was *K* = 2. According to the clustering results, the samples were subjected to single-sample gene set enrichment analysis (ssGSEA) using “GSVA” package [14]. Immune function gene set and relative abundance of immune cells of each sample were analyzed according to the immune gene set obtained in the study by Bindea et al. [15]. According to immune level of samples, samples were grouped into high- and low-immune subtypes, “survival” package [16] was employed to plot the survival curves of high- and low-immune subtypes, and differences in survival time between two groups were measured by log-rank test.

### 2.3. Screening of Prognostic Relevant Differentially Expressed Immune Genes

HCC data were processed (data were converted from FPKM format to TPM format), and immune genes of high- and low-immune subtypes were analyzed for differential expression (|log*FC*| > 2, *P*.adj < 0.05) using “limma” package [17], with high-immune subtype as control, to obtain immune-related differentially expressed genes (IRGs) of HCC. GO and KEGG enrichment analyses were conducted using “clusterProfiler” package [18], and results were visualized using the “enrichplot” package (CRAN-Package shadowtext (http://r-project.org/).

### 2.4. Establishment and Evaluation of Immune Gene-Related Prognostic Risk Assessment Model

Data from the TCGA-LIHC dataset, samples were randomly assigned to training set (*n* = 297) and validation set (*n* = 127) at a 7 : 3 ratio. Combined with the survival information of the training set (follow − up time > 30 days), “survival” package was utilized for univariate Cox regression analysis of IRGs (*P* < 0.01) to obtain IRGs related to survival. Lasso regression was performed on the IRGs screened by univariate regression analysis using the “glmnet” package [19]. Finally, “survminer” package was utilized to perform the multivariate Cox regression model on genes screened by lasso to obtain prognostic-related genes and construct a risk assessment model.

(1) $$\begin{array}{c} \text{RiskScore}=\overset{n}{\underset{i=1}{\sum }}exp_{i}*\beta_{i}, \end{array}$$

where *n* is the number of genes screened to be characteristic of prognosis, exp*_i_* is the expression value of each prognostic key gene, and *β_i_* is the corresponding multivariate Cox regression coefficient.

The median risk score was utilized as a cutoff value to classify patient samples into high-risk and low-risk groups, and *K*-*M* survival curves were drawn using the “survival” package. Receiver operating characteristic (ROC) curve was plotted using “survivalROC” package [20], area under the curve (AUC) values of 3-year and 5-year overall survival (OS) were calculated, and then model was validated using the validation set, so as to measure the predictive ability of the model.

### 2.5. Nomogram Construction and Evaluation

Univariate and multivariate Cox regression analyses were conducted by combining the risk scores with clinical factors (age, gender, T, N, M) to verify the independence of the model. A nomogram was plotted using “rms” package [21] combining clinical information with risk scores to predict likelihood of survival of HCC patients at 3 and 5 years, using “foreign” package (https://cran.r-project.org/web/packages/foreign/index.html). Then, calibration curves of nomogram for 3 and 5 years were generated to assess the predictive effect of the nomogram.

### 2.6. Assessment of Immune Cell Infiltration

The TIMER database, a database that detects the immune cell infiltration in tumor tissues combined with RNA-Seq expression profiling data, can provide the immune infiltration levels of six immune cells (B cells, CD4^+^ T cells, CD8^+^ T cells, neutrophils, macrophages, and dendritic cells) in tumors. In this study, based on the TIMER database, the selected prognosis-related immune genes were analyzed to evaluate correlation between immune genes and infiltration levels of six immune cells.

### 2.7. Cell Culture and Transfection

Human monocytic THP-1 cells (BNCC100407, China) were differentiated into macrophage-adherent like cells using 10 ng/ml Phorbol 12-myristate 13-acetate (Sigma, USA). Subsequently, hepatoma cells HepG2 cells (BNCC338070, China) and THP-1 were maintained in a medium containing RPMI-1640+10% FBS with 5% CO_2_ at 37°C. A blank pcDNA3.1 plasmid (oe-NC) vector (Ribobio, China) was purchased to construct plasmid vectors for pcDNA3.1-NR0B1 (oe-NR0B1) and pcDNA3.1-FGF9 (oe-FGF9). The pcDNA3.1-NR0B1 plasmid, pcDNA3.1-FGF9 plasmid, and corresponding blank pcDNA3.1 plasmid were transfected into the hepatoma cell line HepG2 using Lipofectamine 2000 kit (Invitrogen, USA). After 24 h, the transfected cells were used for the next experiment.

### 2.8. qRT-PCR

RNA isolation was conducted with reference to method of Xia et al. 22]. Total RNA from cells was analyzed by qRT-PCR using an ABI 7500 Real-Time Quantitative PCR System (AB, USA) with GAPDH as an internal reference. Finally, relative quantification was computed using the 2^-*ΔΔ*Ct^ method. Primer sequences utilized for PCR are shown in Table 1.

### 2.9. Transwell Assay

Macrophages (2 × 10^5^ cells/ml) were added to the upper chamber (Corning, USA). HepG2 cells stably transfected with oe-NR0B1 and oe-FGF9 were supplemented to the lower chamber and cultured with DMEM plus 10% FBS. They were then cultured for 8 h under routine conditions, after which cells in the upper chamber that had not crossed the membrane were removed, fixed with 4% paraformaldehyde solution for 15 min, and stained with 0.1% crystal violet for 15 min. Five fields were randomly selected to analyze invasion rate of macrophages under a light microscope (×100), and the experiment was performed in triplicate [22, 23].

### 2.10. Data Analysis

Data analysis was mainly conducted using *R* software, GraphPad Prism 6 software (GraphPad Software, USA). For bioinformatics analysis, statistical test was conducted using the statistical test method corresponding to the *R* package, and statistical significance was considered when *P* < 0.05. For macrophage migration assay and qPCR assay, all results were presented as mean ± SD. The differences were compared using *t*-test, with *P* < 0.05 suggesting statistically significant differences.

## 3. Results

### 3.1. Prognostic Analysis of Different Immune Gene Subclusters

**Figure 1:**
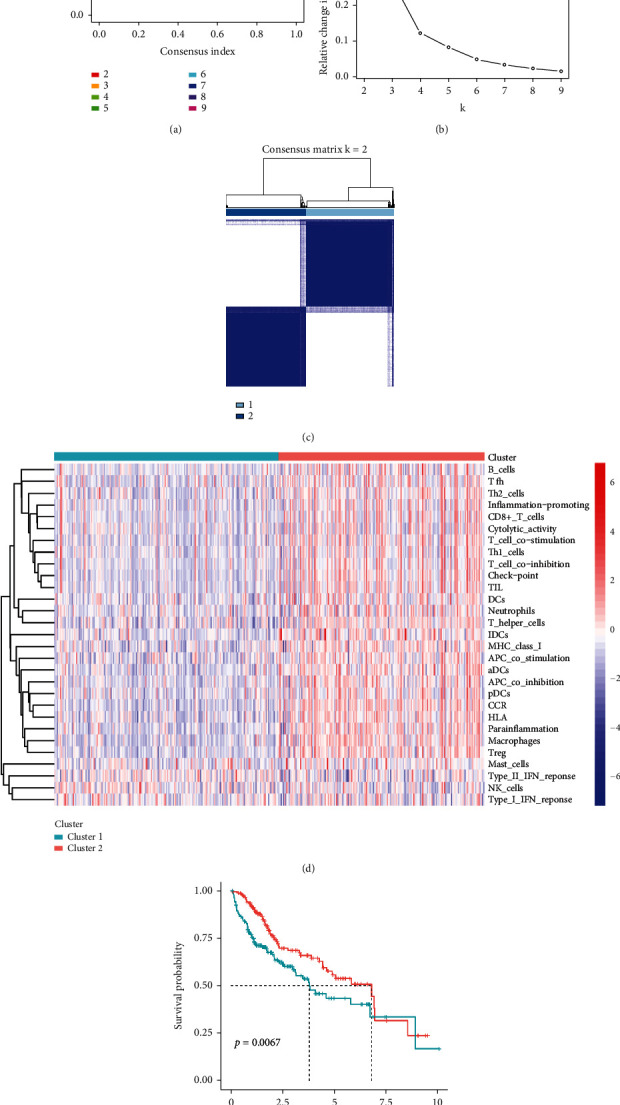
Consensus clustering and survival analysis of HCC based on hepatocyte immune-related gene sets. (a) CDF curves with different *K* values. (b) CDF delta area plot, which represents relative change in the area under the CDF curve for *k* compared to *k* − 1, with abscissa indicating *k* and ordinate indicating relative change in the area under the CDF curve. (c) 374 HCC patients were divided into 2 molecular subtypes. (d) Heatmap of 29 immune gene expression levels in samples with 2 molecular subtypes. (e) Kaplan-Meier survival curves between high- and low-immune subtypes.

Cluster analysis of immune-related gene sets of HCC was performed for determining HCC immune subtypes. The results of the consensus clusters were visualized by using plots of the empirical cumulative distribution function (CDF) and CDF delta area, where *K* denotes the number of isoforms (Figures 1(a) and 1(b)). The results showed that when *K* = 2, the internal consistency of the clusters was high, and the clustering worked best (Figure 1(c)). The 374 tumor patients with clinical information were therefore divided into 2 clusters, including 195 in cluster 1 and 179 in cluster 2. Combined with ssGSEA results, it could be seen that cluster 1 had a low immune level and was therefore defined as a low-immune subtype. The cluster 2 had a high degree of immunity and was defined as a high-immune subtype (Figure 1(d)). Survival difference between two groups was significant because *P* value of survival analysis was less than 0.05 (Figure 1(e)).

**Figure 2:**
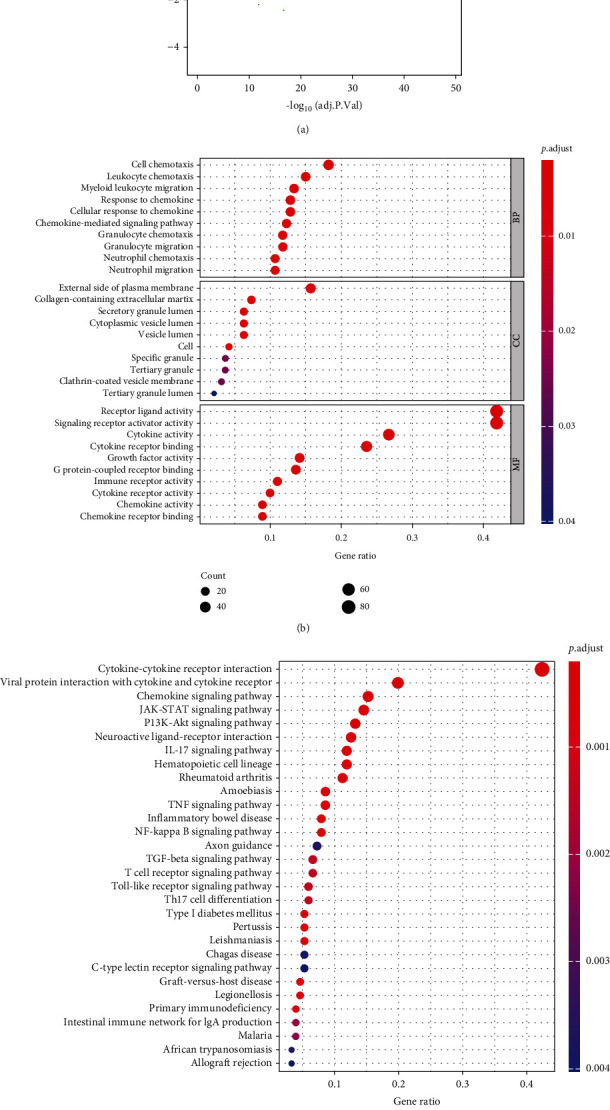
Differential expression analysis of immune genes as well as enrichment analyses. (a) Volcano plot of IRGs of HCC. Red: markedly upregulated genes; green: markedly downregulated genes. GO (b) and KEGG (c) enrichment analyses bubble plot of IRGs.

Totally 192 differentially expressed IRGs were screened out, including 190 upregulated genes and 2 downregulated genes (Figure 2(a)). GO analysis illustrated that differential genes were mainly enriched in biological functions such as cell chemotaxis (Figure 2(b)). KEGG enrichment analysis showed that differential genes were mainly gathered in immune-related signaling pathways such as cytokine-cytokine receptor interaction, chemokine signaling pathway, and JAK-STAT signaling pathway (Figure 2(c)).

### 3.2. Screening of Immune-Prognostic Genes and Validation of Prognostic Model in HCC

**Figure 3:**
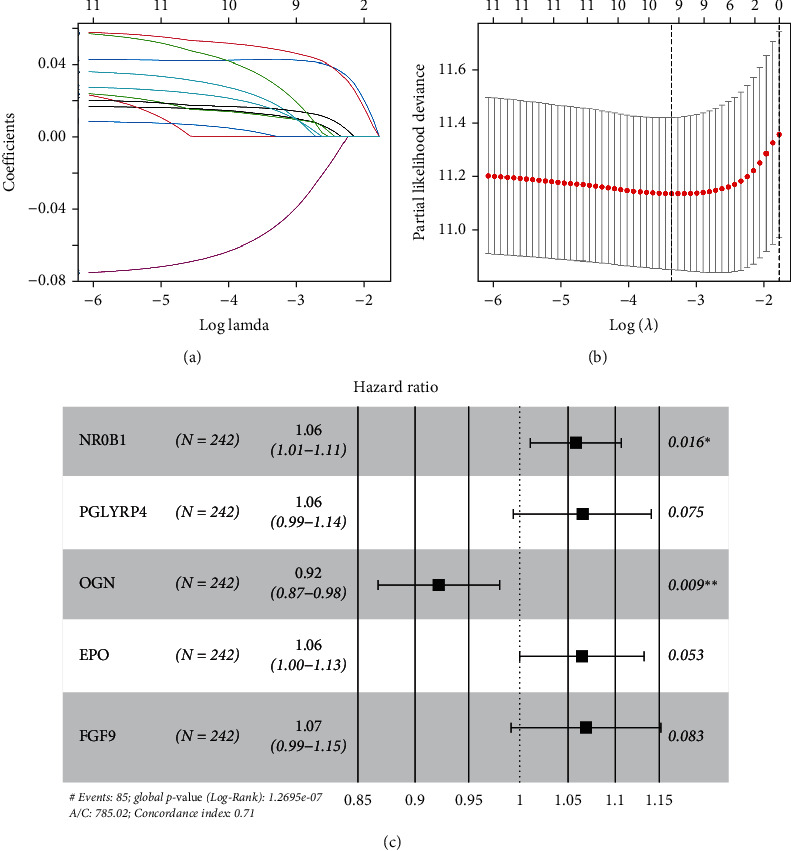
Immunological profile prognostic model construction. (a) The coefficients of the 12 prognostication-related IRGs in the lasso analysis change trajectory with the penalty function lambda. (b) The selection interval of the optimal penalty parameter, and the upper coordinate represents the number of genes at different lambda values. (c) Forest plot of multivariate Cox analysis of five prognosis-related IRGs (^∗^*P* < 0.05, ^∗^*P* < 0.01).

Univariate Cox regression analysis of IRGs using “survival” package (*P* < 0.01) selected 12 IRGs associated with HCC prognosis (Table S2). Subsequent lasso regression analysis of these 12 prognostic relevant genes resulted in the selection of nine IRGs that were significantly associated with prognosis (Figures 3(a) and 3(b); Table S3). Multivariate Cox analysis of the genes selected by lasso finally resulted in five optimal prognostic IRGs (NR0B1, PGLYRP4, OGN, EPO, and FGF9). From the forest plot of the prediction model, it could be seen that the hazard ratio (HR) was greater than 1 for genes NR0B1, PGLYRP4, EPO, and FGF9, and the HR value was less than 1 for gene OGN (Figure 3(c); Table S4).

### 3.3. Prognostic Ability Assessment of 5-Gene Model

**Figure 4:**
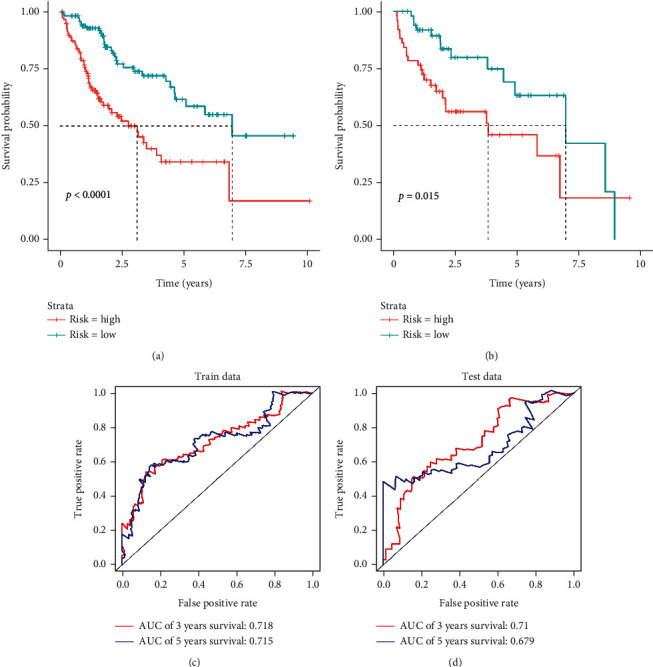
5-Gene model prediction ability assessment. *K*-*M* survival curves of patients in the high- (red) and low-risk (green) groups in training set (a) and validation set (b). (c) ROC curves for the training set 5-gene prognostic model. (d) ROC curves for the validated set 5-gene prognostic model.

**Figure 5:**
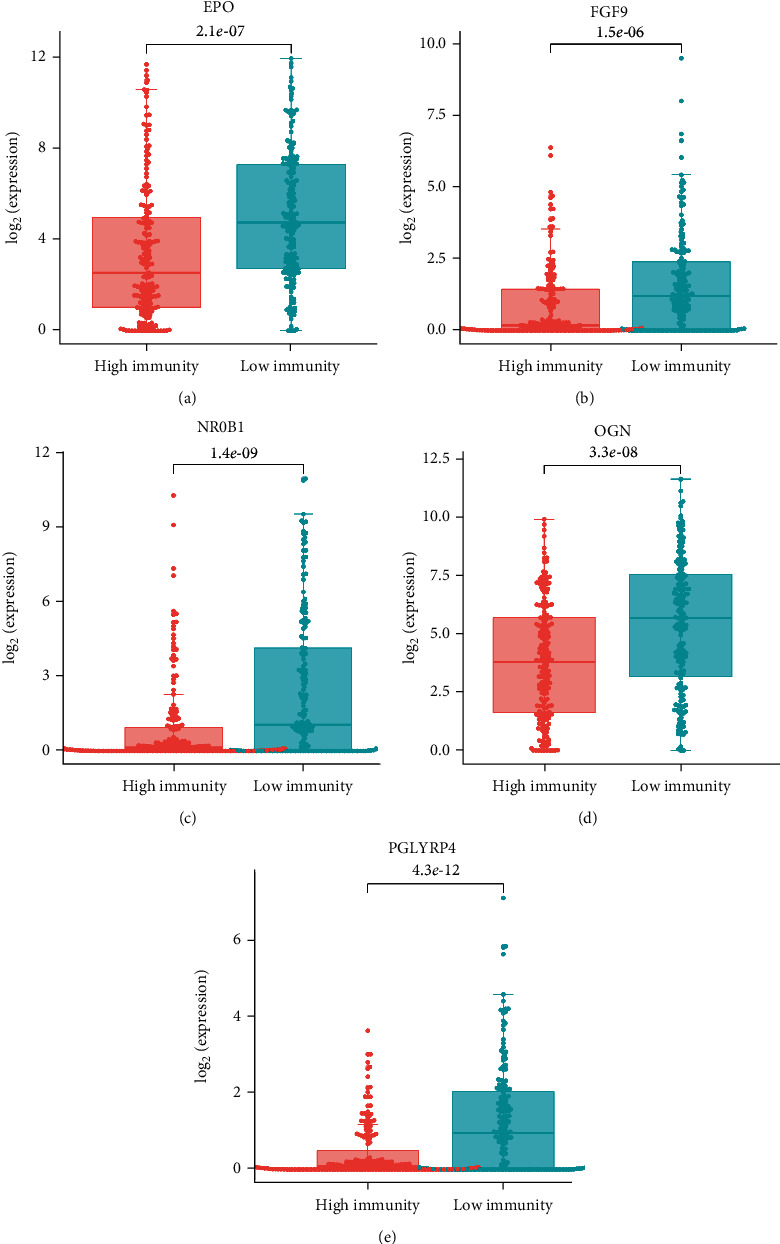
Box plot of expression levels of 5 genes between high- and low-immune subtypes. EPO (a), FGF9 (b), NR0B1 (c), OGN (d), and PGLYRP4 (e) levels between high- and low-immune subtypes.

The *K*-*M* survival curve of patients in high- and low-risk groups was plotted. It was showed that survival of the high-risk group presented noticeably lower survival rate than the low-risk group as the risk score elevated (Figure 4(a)). *K*-*M* curves of the validation set showed the same result as in the training set (Figure 4(b)). To further assess predictive effect of the 5-gene model, ROC curve plotting was performed using the “SurvivalROC” package, which showed that the AUC values of 3-year and 5-year survival of patients in the training set were 0.718 and 0.715, respectively (Figure 4(c)), while the AUC values were 0.710 and 0.679 for the 3- and 5-year survival of patients in validation set, respectively (Figure 4(d)). Finally, differential distribution analysis exhibited that the expression levels of the five characteristic genes in the high-immune group were significantly higher than those in the low-immune group (Figures 5(a)–5(e)). The above results demonstrated that risk score of the 5-gene model constructed in this study had some predictive power for HCC patients.

### 3.4. Validation of Independence of the 5-Gene Model

**Figure 6:**
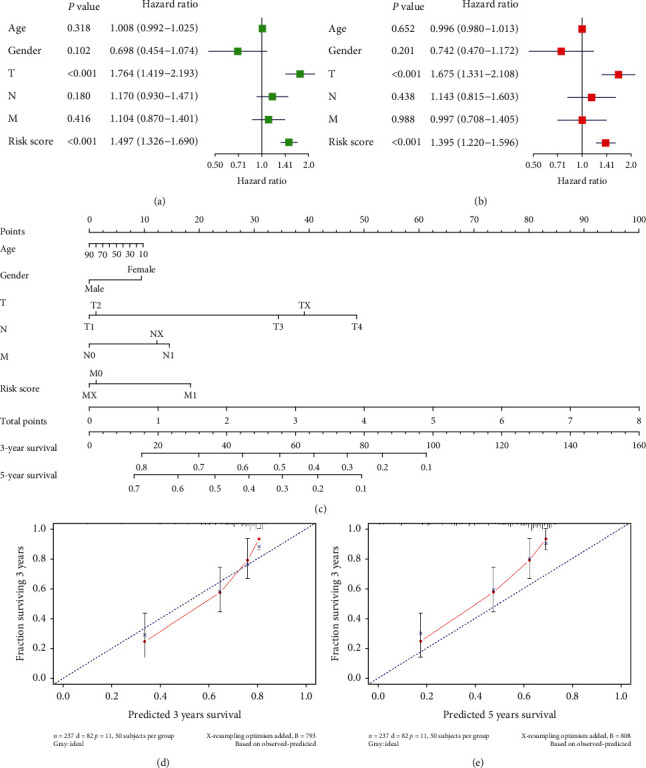
Correlation analysis of risk scores and clinical factors with the prognosis of HCC patients and construction and assessment of nomograms. Forest plots of univariate Cox regression analysis (a) and multivariate Cox regression analysis (b) of risk score combined with clinical factors. (c) Nomogram of risk score combined with clinical factors to predict 3- and 5-year survival of patients; calibration curve for nomogram prediction of 3-year survival (d) and 5-year survival (e) of HCC patients.

Univariate Cox regression analysis of 5-gene risk score and other clinical factors (age, gender, T, N, M) showed that risk score and T stage were highly significant (*P* < 0.01), demonstrating that these two factors, risk score and T stage, had an impact on prognosis (Figure 6(a)). Moreover, multivariate Cox regression analysis result presented that risk score and T stage were significant (*P* < 0.05). Altogether, risk score obtained from the immune-related 5-gene risk assessment model could be used as an independent prognostic factor for HCC (Figure 6(b)). Subsequently, in order to predict the 3- and 5-year survival rates of HCC patients, we constructed a prognostic nomogram of HCC patients combining risk score with clinical factors (age, gender, T, N, M) and plotted calibration curve at 3 and 5 years. The calibration curve showed good fitting of the characteristics, indicating that nomogram can well predict survival of patients. Comprehensive analysis suggested that the model could effectively predict prognosis of HCC patients (Figures 6(c)–6(e)).

### 3.5. Immune-Related Genes and Immune Cell Infiltration Assessment

**Figure 7:**
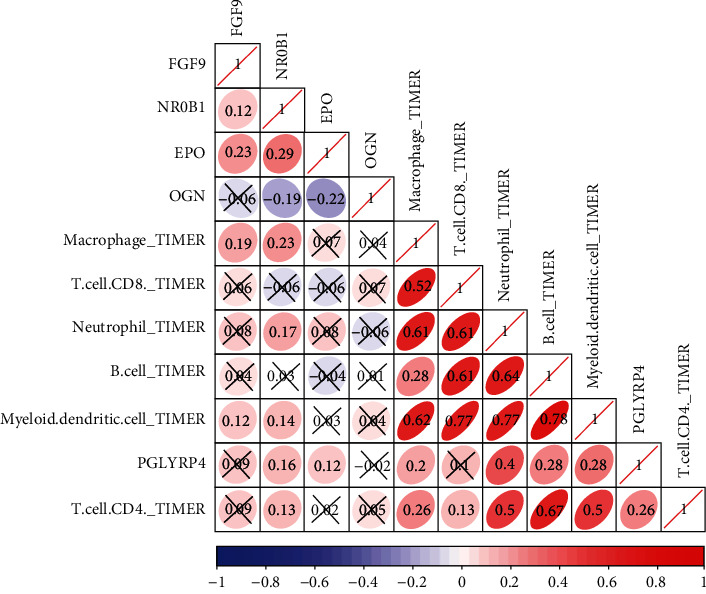
Immune cell infiltration analysis (TIMER). Red refers to positive correlation, blue refers to negative correlation, and the darker the color, the stronger the correlation. *X* represents that the correlation results between genes and immune cells are not significant.

Correlation analysis of immune-related genes and immune cell infiltration was performed based on the TIMER database. The results showed that NR0B1 was notably correlated with macrophage (*r* = 0.23), neutrophils (*r* = 0.17), myeloid dendritic cells (*r* = 0.14), and CD4^+^ T cells (*r* = 0.13). FGF9 was correlated with macrophage (*r* = 0.19) and myeloid dendritic cells (*r* = 0.12), and all were positively correlated (Figure 7). These results reflect the potential of NR0B1 and FGF9 to influence the tumor immune microenvironment.

### 3.6. Immune-Related Genes Promote Macrophage Chemotaxis

**Figure 8:**
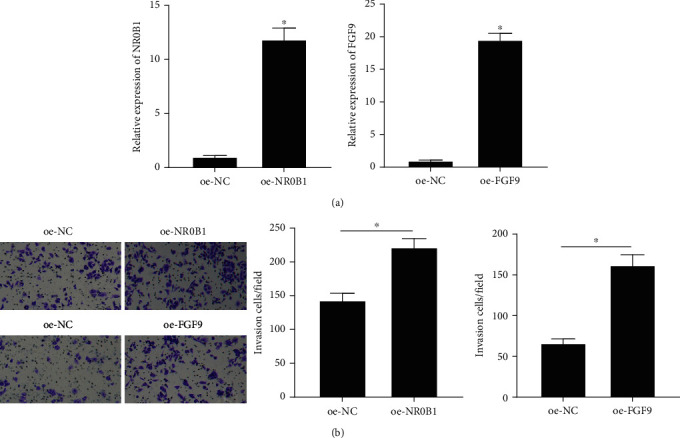
Effects of immune-related genes on macrophage chemotaxis. (a) The transfection efficiency of oe-NR0B1 and oe-FGF9 plasmids. (b) The effect of hepatoma cells overexpressing NR0B1 and FGF9 on macrophage chemotaxis (^∗^*P* < 0.05).

To investigate the relationship between immune-related genes NR0B1 and FGF9 and macrophage chemotaxis, NR0B1 and FGF9-overexpressing HepG2 cells were constructed, respectively, in this study. The detection of mRNA expression levels of NR0B1 and FGF9 in HepG2 cells using qRT-PCR showed that NR0B1 and FGF9 were significantly increased in the oe-NR0B1 and oe-FGF9 groups, respectively (Figure 8(a)), and could be used for the next experimental study. Transwell assay was conducted to examine effects of NR0B1 and FGF9 on macrophage chemotaxis and revealed that when NR0B1 and FGF9 were overexpressed in HCC cells, the number of macrophage migration was remarkably upregulated (Figure 8(b)), indicating that the overexpression of NR0B1 and FGF9 could enhance macrophage chemotaxis to tumor tissues.

## 4. Discussion

Although the medical level has been continuously improving and the understanding of HCC has been deepening, incidence and mortality rates of HCC remain high, and the prognosis is not optimistic. Therefore, accurate prognosis estimation of patients with HCC is essential for subsequent treatment of patients. Researchers have found that the patient's immune system can be modified to recognize specific antigens on cancer cells and enhance immune activity by blocking the immune checkpoints responsible for immunosuppressive signals [24]. Therefore, screening biomarkers related to immune genes is pivotal for patient's prognosis and targeted therapy of HCC.

Herein, we analyzed IRGs by grouping the immune genes of HCC, selected five IRGs (NR0B1, PGLYRP4, OGN, EPO, and FGF9) associated with prognostic characteristics, and constructed a 5-gene risk assessment model. NR0B1 is mainly expressed in the adrenal cortex, ovary, and Sertoli cells, and it has been found that NR0B1 (also known as DAX-1) can suppress proliferation of HCC cells by regulating transcriptional activity of *β*-catenin [25]. OGN can inhibit breast cancer cell proliferative and invasive properties via mediating PI3K/Akt/mTOR signaling pathway [26]. It has been demonstrated that EPO enhances self-renewal and expansion ability of cancer stem cells. Previous study illustrated therapeutic potential of blocking EPO/EPOR/JAK/STAT signaling in HCC patients with polycythemia [27]. FGF9 can enhance the tumor-forming ability and resistance to sorafenib in HCC cells, and forced expression of FGF9 is associated with dismal prognosis in HCC patients [28]. In comprehensive analysis, the immune-related genes have significant potential in the clinical treatment of solid tumors. Therefore, the immune-related genes of HCC are expected to be prognostic markers for HCC.

To verify the relationship between immune-related genes screened in this study and immune cells, we evaluated infiltration levels of immune cells and immune-related genes and selected two genes, NR0B1 and FGF9, that best correlated with immune cells. The effects of NR0B1 and FGF9 on macrophage migration were verified, and we found that the overexpression of NR0B1 and FGF9 could enhance macrophage migration. Several studies have demonstrated dual function of macrophages in HCC, and M2 macrophages are generally considered to be protumor, while M1 macrophages are considered to be antitumor [29]. It has been demonstrated that the degree of macrophage infiltration is high in HCC, and prognosis of patients is not good [30]. Analysis results of immune-related genes in HCC showed that NR0B1 and FGF9 were risk factors for HCC, and experiments confirmed that NR0B1 and FGF9 could enhance the migration ability of macrophages. The study by Chang et al. confirmed that FGF9 can enhance the immune infiltration ability of M2 macrophages [31]. KEGG enrichment analysis demonstrated that IRGs were mainly enriched in immune-related pathways such as chemokine signaling pathway, which can confirm that the prognostic model constructed according to immune-related genes of HCC in this study has high reliability.

This study has some shortcomings. First, the analysis is based only on the data in the public database, and the constructed model is also based only on the public database, which is not validated and needs to be validated in combination with clinical sample information. Secondly, in this study, the genes of HCC were screened by *K*-means consistency clustering after HCC patients were classified as high- and low-immune subtypes. The obtained prognostic markers of immune-related HCC had a high correlation with nonspecific immune cells and a low degree of correlation with specific immune cells. Although cell experiments were performed for bioinformatics results, this was only limited to the laboratory. Subsequently, clinical research is needed to confirm results of this study.

In summary, we grouped immune-related genes of HCC, obtained five immune-related genes related to prognosis according to the screening of immune-related genes of HCC, and validated a prognostic risk assessment model for HCC. And risk score obtained from the model constructed in this study can be utilized as an important prognostic factor independent of clinical characteristics. The screened immune-related genes are likely to be potential targets for HCC, providing some theoretical basis for the prognosis of HCC and the development of personalized diagnosis and treatment plan.

## Figures and Tables

**Table 1:** Primer sequences for qRT-PCR.

Gene	Sequence	
NR0B1	Forward primer	5′-TCCGCGCCCTTGCCCAGACC-3′
	Reverse primer	5′-GCCGCACGAACAGCCCCAA-3′
FGF9	Forward primer	5′-CCAGGACTAAACGGCACCAGAA-3′
	Reverse primer	5′-AATAAGAACCCACCGCATGAAAG-3′
GAPDH	Forward primer	5′-ATGACATCAAGAAGGTGGTG-3′
	Reverse primer	5′-CATACCAGGAAATGAGCTTG-3′

## Data Availability

The data used to support the findings of this study are included within the article. The data and materials in the current study are available from the corresponding authors on reasonable request.

## Supplementary Materials

Supplementary 1 Table S1: immune-related genes of HCC.

Supplementary 2 Table S2: genes selected by univariate Cox regression analysis.

Supplementary 3 Table S3: genes selected by lasso regression analysis.

Supplementary 4 Table S4: genes selected by multivariate Cox regression analysis.
