# Supplementary material for: Prediction of Prognosis in Patients with Hepatocellular Carcinoma Based on Molecular Subtypes of Immune Genes
Source: Gastroenterol Res Pract. 2022 Jun 28;2022:2746156. doi: 10.1155/2022/2746156 (PMC9274231; doi:10.1155/2022/2746156)
Supplement: Supplementary 2 — Table S2: genes selected by univariate Cox regression analysis. [file 2746156.f2.pdf]

id	HR	HR.95L	HR.95H	pvalue
NR0B1	1.107711	1.062523	1.15482	1.48E-06
EPO	1.141248	1.075675	1.210817	1.21E-05
PAEP	1.074152	1.034909	1.114883	0.000165
FABP6	1.08826	1.037017	1.142034	0.000588
SPP1	1.110571	1.045176	1.180058	0.000707
S100A2	1.162541	1.064477	1.269639	0.000809
OGN	0.907371	0.855712	0.962149	0.001153
FGF9	1.114883	1.036275	1.199453	0.003555
AQP9	0.900643	0.838087	0.967868	0.004383
NPPB	1.071858	1.019151	1.12729	0.00699
EGF	1.073549	1.01958	1.130374	0.007001
PGLYRP4	1.093529	1.023698	1.168124	0.007916
