# Supplementary material for: Prediction of Prognosis in Patients with Hepatocellular Carcinoma Based on Molecular Subtypes of Immune Genes
Source: Gastroenterol Res Pract. 2022 Jun 28;2022:2746156. doi: 10.1155/2022/2746156 (PMC9274231; doi:10.1155/2022/2746156)
Supplement: Supplementary 3 — Table S3: genes selected by lasso regression analysis. [file 2746156.f3.pdf]

Gene ID
SPP1
S100A2
NR0B1
PGLYRP4
OGN
PAEP
EPO
FGF9
NPPB
