# Supplementary material for: Prediction of Prognosis in Patients with Hepatocellular Carcinoma Based on Molecular Subtypes of Immune Genes
Source: Gastroenterol Res Pract. 2022 Jun 28;2022:2746156. doi: 10.1155/2022/2746156 (PMC9274231; doi:10.1155/2022/2746156)
Supplement: Supplementary 4 — Table S4: genes selected by multivariate Cox regression analysis. [file 2746156.f4.pdf]

id	coef	HR	HR.95L	HR.95H	pvalue
NR0B1	0.055905	1.057497	1.010486	1.106695	0.015972
PGLYRP4	0.062779	1.064792	0.993673	1.141	0.075075
OGN	-0.08149	0.921739	0.867427	0.979453	0.008539
EPO	0.061897	1.063853	0.999322	1.132551	0.052535
FGF9	0.06637	1.068622	0.991314	1.151959	0.083227
